# Intelligent Bayes Classifier (IBC) for ENT infection classification in hospital environment

**DOI:** 10.1186/1475-925X-5-65

**Published:** 2006-12-18

**Authors:** Ritaban Dutta, Ritabrata Dutta

**Affiliations:** 1 University of Reading, Reading RG6 6AY, UK; 2 Indian Statistical Institute, Kolkata 700108, India

## Abstract

Electronic Nose based ENT bacteria identification in hospital environment is a classical and challenging problem of classification. In this paper an electronic nose (e-nose), comprising a hybrid array of 12 tin oxide sensors (SnO_2_) and 6 conducting polymer sensors has been used to identify three species of bacteria, *Escherichia coli *(*E. coli*), *Staphylococcus aureus *(*S. aureus*), and *Pseudomonas aeruginosa *(*P. aeruginosa*) responsible for ear nose and throat (ENT) infections when collected as swab sample from infected patients and kept in ISO agar solution in the hospital environment. In the next stage a sub-classification technique has been developed for the classification of two different species of S. aureus, namely Methicillin-Resistant S. aureus (MRSA) and Methicillin Susceptible S. aureus (MSSA). An innovative Intelligent Bayes Classifier (IBC) based on "Baye's theorem" and "maximum probability rule" was developed and investigated for these three main groups of ENT bacteria. Along with the IBC three other supervised classifiers (namely, Multilayer Perceptron (MLP), Probabilistic neural network (PNN), and Radial Basis Function Network (RBFN)) were used to classify the three main bacteria classes. A comparative evaluation of the classifiers was conducted for this application. IBC outperformed MLP, PNN and RBFN. The best results suggest that we are able to identify and classify three bacteria main classes with up to 100% accuracy rate using IBC. We have also achieved 100% classification accuracy for the classification of MRSA and MSSA samples with IBC. We can conclude that this study proves that IBC based e-nose can provide very strong and rapid solution for the identification of ENT infections in hospital environment.

## 1. Background

An electronic nose (e-nose) is an instrument that has been developed as a simplified "electronic" model of the human olfactory system. To humans, the sensation of flavour is due to three main chemoreceptor systems. These are gustation (sense of taste by tongue), olfaction (sense of smell by nose) and trigeminal (sense of irritation of trigeminal receptors). The sense of taste is used to detect certain non-volatile chemicals, which enter the mouth while the sense of smell is used to detect certain volatile compounds. Receptors for the trigeminal sense are located in the mucous membranes and in the skin, they respond to certain volatile chemicals and it is thought to be especially important in the detection of irritants and chemically reactive species. In the perception of flavour all three chemoreceptor systems are involved but olfaction plays by far the greatest role. An electronic nose (e-nose) is an instrument that is designed to detect and discriminate different complex odours using a sensor array. The sensor array consists of broadly tuned (non-specific) sensors that are treated with a variety of odour-sensitive chemical materials [1,2].

An odour stimulus generates a characteristic fingerprint (or smell-print) from the sensor array. Patterns, or fingerprints, from known odours are then used to construct a database and train a pattern recognition system so that unknown odours can subsequently be classified, i.e. identified. Thus, e-noses comprise of mechanical components to collect and transport odours to the sensor array as well as electronic circuitry to digitize and store the sensor responses for signal processing.

Gardner and Bartlett defined an electronic nose as "An instrument, which comprises an array of electronic chemical sensors with partial specificity and an appropriate pattern recognition system, capable of recognizing simple or complex odours". The EN system is designed for automated detection and classification of odours, vapours, and gases. It can also perform simple odour discrimination and provide measurement of odour intensity. The two main components of an e-nose are the sensing system and the automated pattern recognition system [3,4].

SAW (surface acoustic wave) and QMB (quartz micro balances piezo crystals) both utilize common GC stationary phases to absorb odorant molecules. Both suffer from a lack of sensitivity. CP (conducting polymers) can be very specific but are very sensitive to moisture, which makes them difficult to use in food and beverage analysis. MOS (metal oxide sensors) are the most sensitive and stable of the sensor technologies applied. They have emerged as the bench mark of stable reproducible instruments.

In this paper we describe the use of a hybrid sensors based e-nose [5] for illness diagnosis. The system consists of 12 tin oxide sensors (SnO2) and 6 conducting polymer sensors configured into a hybrid array has been used to identify three species of bacteria, Escherichia coli (E. coli), Staphylococcus aureus (S. aureus), and Pseudomonas aeruginosa (P. aeruginosa) responsible for ear nose and throat (ENT) infections when collected as swab sample from infected patients and kept in ISO agar solution in the hospital environment. In the next stage a sub-classification technique has been developed for the classification of two different species of S. aureus, namely Methicillin-Resistant S. aureus (MRSA) and Methicillin Susceptible S. aureus (MSSA) [1].

## 2. ENT bacteria for this study

### 2.1. Escherichia coli

Escherichia coli, usually abbreviated to E. coli, discovered by Theodor Escherich, a pediatrician and bacteriologist, is one of the main species of bacteria that live in the lower intestines of warm-blooded animals, including birds and mammals. They are necessary for the proper digestion of food and are part of the intestinal flora. Its presence in groundwater is a common indicator of fecal contamination. The name comes from its discoverer, Theodor Escherich. It belongs among the Enterobacteriaceae, and is commonly used as a model organism for bacteria in general. One of the root words of their family's scientific name, "enteric", refers to the intestine, hence "gastroenteritis" (from 'gastro-', stomach, 'entero-' intestine, '-itis', disease). "Fecal" is the adjective for organisms that live in feces, so it is often used synonymously with "enteric" [6].

The number of individual E. coli bacteria in the feces that one human passes in one day averages between 100 billion and 10 trillion. All the different kinds of fecal coli bacteria and all the very similar bacteria that live in the ground (in soil or decaying plants, of which the most common is Enterobacter aerogenes are grouped together under the name coliform bacteria. Technically, the "coliform group" is defined to be all the aerobic and facultative anaerobic, non-spore-forming, Gram-negative, rod-shaped bacteria that ferment lactose with the production of gas within 48 hours at 35°C (95°F). In the body, this gas is released as flatulence).

### 2.2. Pseudomonas aeruginosa

Pseudomonas aeruginosa is an opportunistic pathogen that usually causes problems in humans who have weakened immune systems. This bacterium usually infects the urinary tract, burns, wounds, and also causes other blood infections. One in ten hospital acquired infections is from Pseudomonas. Cystic fibrosis patients are also predisposed to P. aeruginosa infection of the lungs. P. aeruginosa is also the typical cause of "hot-tub rash" (dermatitis), caused by lack of proper, periodic attention to water cleanliness maintenance procedures. This species is also known to be an opportunistic pathogen of plants [6].

### 2.3. Staphylococcus aureus

Staphylococcus aureus (which is occasionally given the nickname golden staph) is a bacterium, frequently living on the skin or in the nose of a healthy person, that can cause illnesses ranging from minor skin infections (such as pimples, boils, and cellulitis) and abscesses, to life-threatening diseases such as pneumonia, meningitis, endocarditis and septicemia. For example each year some 500,000 patients in American hospitals contract a staphylococcal infection. It is a spherical bacterium [6,7].

Staphylococcus lives as a commensal on the skin and in the nose of humans and other animals, as well as in the environment. It can infect other tissues when normal barriers have broken down (e.g. skin or mucosal lining). This leads to furuncles (boils) and carbuncles (a collection of furuncles). In infants Staphylococcus aureus infection can cause a severe disease SSSS (staphylococcal scalded skin syndrome).

Staphylococcal infections can be spread through contact with pus from an infected wound, skin to skin contact with an infected person, and contact with objects such as towels, sheets, clothing, or athletic equipment used by an infected person.

Deep Staphylococcus infections can be very severe. Prosthetic joints are particularly at risk, and staphylococcal endocarditis (infection of the heart valves) and pneumonia may be rapidly fatal [8].

### 2.4. MRSA and MSSA

Methicillin-resistant Staphylococcus aureus (MRSA) is a subgroup within a group of organisms known as S. aureus. MRSA are characterized by their resistance to treatment with commonly used antibiotics, in contrast to the remainder of the Staphyloccocus aureus group which are referred to as methicillin-susceptible S. aureus (MSSA). Both MRSA and MSSA can cause infection but individuals may also carry the organism without being infected by it. An individual, who carries the organism, but is not infected, is said to be a 'carrier' or 'colonized'. At any one time up to 33% of healthy individuals carry Staphylococcus aureus, including MRSA, predominantly in their noses and also at other sites. Methicillin Resistant Staphylococcus aureus, MRSA, is regarded as the hospital 'superbug' by virtue of its ability to spread and cause outbreaks with a high mortality rate of up to 60%.

Staphylococcus aureus can give rise to infections varying from mild, e.g. boils and infected cuts, to severe, e.g. infections of bones, lungs, heart and blood stream. The difficulty in treating MRSA with antibiotics has led to concern about this particular group of staphylococcal infections. All strains of Staphyloccocus aureus, including MSSA and MRSA, are capable of causing hospital-acquired infection [6,9,10].

Organisms can be passed to patients from contact with hands or directly from the environment. The latter includes air, dust, clothing, soft furnishings, surfaces and equipment.

Some patients are more susceptible to colonization and infection than others. These include the elderly, patients with wounds, ulcers or bedsores, catheterized patients, those who have received antibiotics and those who have been, or who are, hospitalized or institutionalized.

## 3. Experiment & data gathering

### 3.1 Swab samples

Swab samples were collected from the ENT patients who were suffering from these bacteria infections. The infected swab sample sniffing experiments were conducted using a tin oxide sensors based e-nose at the Heartland hospital, Birmingham, UK. The Heartland hospital, Birmingham, UK ethic committee approved this study [1,10,11]. With the assistance of two ENT specialists in Heartlands hospital swab samples from three types of patients' swab samples were collected. These three types of samples were as follows:

**• *Sample Type 1: ***Swab samples representing Escherichia coli. Total no. of real patient swab collected was 120.

**• *Sample Type 2: ***Swab samples representing Pseudomonas aeruginosa. Total no. of real patient swab collected was 150.

**• *Sample Type 3: ***Swab samples representing Methicillin Resistant S. aureus (MRSA). Total no. of real patient swab collected was 100.

**• *Sample Type 4: ***Swab samples representing Methicillin Susceptible S. aureus (MSSA). Total no. of real patient swab collected was 60.

Sample type 3 and 4 were colleted as part of S. aureus data base. We got 430 swab samples from 430 different patients which covers all 4 types of bacterial class and infections. Samples were sniffed following same procedure.

### 3.2. Test procedure

A strategy to collect the ENT odour samples and sampling procedure was agreed with the ENT specialists and was used throughout the data gathering process. Both protocol and sampling technology were kept simple to be cost effective and non-application specific. Swab samples were collected from the infected areas of the ENT patients' ear, nose and throat regions from the clinical patients. After collection, all swab samples were kept in ISO S type agar solution in typical 15 cm^3 ^vials. This vial size was chosen so as to generate sufficient headspace from the bacterial solution for sniffing purposes. All swab samples were kept in the agar solution in a vial to help the growth of the ENT bacteria.

Later we used the electronic nose system to sniff all the samples. We put approximately 3 mg of agar solution of bacteria in a 10 ml vial. We kept the vial containing bacterial solution for 5 min; it was to generate some headspace from bacterial solution. E-nose's inlet was inserted into the vial and headspace was sniffed [1,5,10].

## 4. Data representation & feature extraction

### 4.1 Data representation

Consider an array of *n *discrete sensors, where each sensor *i *produces a time-dependent output signal *x*_*ij*_(*t*) in response to an odour *j*. The electrical sensor signal often depends on several physical parameters (e.g. flow rate, ambient pressure, temperature and humidity), but the sensor outputs will reach constant asymptotic values when presented with a constant input stimulus. It is common practice to use only the static or steady-state values of the sensor signals, the response is then defined simply as, for example, an absolute change in sensor signal. However, the choice of the response parameter is fundamental to the subsequent performance of the pattern recognition technique; the pre-processing technique of the response vectors should be chosen to help analyse data from a specific problem. In order to extract relevant key features from the data in terms of the static change in sensor resistance or conductivity, generally a good choice is using a fractional difference model: xij=(Xijodour−Xio)/Xio
 MathType@MTEF@5@5@+=feaafiart1ev1aaatCvAUfKttLearuWrP9MDH5MBPbIqV92AaeXatLxBI9gBaebbnrfifHhDYfgasaacH8akY=wiFfYdH8Gipec8Eeeu0xXdbba9frFj0=OqFfea0dXdd9vqai=hGuQ8kuc9pgc9s8qqaq=dirpe0xb9q8qiLsFr0=vr0=vr0dc8meaabaqaciaacaGaaeqabaqabeGadaaakeaacqWG4baEdaWgaaWcbaGaemyAaKMaemOAaOgabeaakiabg2da9iabcIcaOiabdIfaynaaDaaaleaacqWGPbqAcqWGQbGAaeaacqWGVbWBcqWGKbazcqWGVbWBcqWG1bqDcqWGYbGCaaGccqGHsislcqWGybawdaqhaaWcbaGaemyAaKgabaGaem4Ba8gaaOGaeiykaKIaei4la8IaemiwaG1aa0baaSqaaiabdMgaPbqaaiabd+gaVbaaaaa@491F@ where Xiodour
 MathType@MTEF@5@5@+=feaafiart1ev1aaatCvAUfKttLearuWrP9MDH5MBPbIqV92AaeXatLxBI9gBaebbnrfifHhDYfgasaacH8akY=wiFfYdH8Gipec8Eeeu0xXdbba9frFj0=OqFfea0dXdd9vqai=hGuQ8kuc9pgc9s8qqaq=dirpe0xb9q8qiLsFr0=vr0=vr0dc8meaabaqaciaacaGaaeqabaqabeGadaaakeaacqWGybawdaqhaaWcbaGaemyAaKgabaGaem4Ba8MaemizaqMaem4Ba8MaemyDauNaemOCaihaaaaa@366C@ is the response of the sensor *i *to the odour sample *j*, and Xio
 MathType@MTEF@5@5@+=feaafiart1ev1aaatCvAUfKttLearuWrP9MDH5MBPbIqV92AaeXatLxBI9gBaebbnrfifHhDYfgasaacH8akY=wiFfYdH8Gipec8Eeeu0xXdbba9frFj0=OqFfea0dXdd9vqai=hGuQ8kuc9pgc9s8qqaq=dirpe0xb9q8qiLsFr0=vr0=vr0dc8meaabaqaciaacaGaaeqabaqabeGadaaakeaacqWGybawdaqhaaWcbaGaemyAaKgabaGaem4Ba8gaaaaa@30D4@ is the corresponding baseline or reference signal, e.g. in ambient room air prior to the odour measurement. The response generated by the *n*-sensor array to an odour *j *can then be represented by a time-dependent vector: *X*(*t*) = (*x*_1*j *_(*t*), *x*_2*j *_(*t*), *K*, *x*_*ij *_(*t*), *K*, *x*_*nj *_(*t*)). When the same array is presented to a set of *m *odours, the response can be regarded as a set of *m *vectors, which are best represented by a response matrix R˜
 MathType@MTEF@5@5@+=feaafiart1ev1aaatCvAUfKttLearuWrP9MDH5MBPbIqV92AaeXatLxBI9gBaebbnrfifHhDYfgasaacH8akY=wiFfYdH8Gipec8Eeeu0xXdbba9frFj0=OqFfea0dXdd9vqai=hGuQ8kuc9pgc9s8qqaq=dirpe0xb9q8qiLsFr0=vr0=vr0dc8meaabaqaciaacaGaaeqabaqabeGadaaakeaacuWGsbGugaacaaaa@2DE8@(*t*):

R˜(t)=(x11(t)x12(t)Kx1m(t)x21(t)x22(t)Kx2m(t)MMO xij(t)Mxn1(t)xn2(t)KO xnm(t))     (1)
 MathType@MTEF@5@5@+=feaafiart1ev1aaatCvAUfKttLearuWrP9MDH5MBPbIqV92AaeXatLxBI9gBaebbnrfifHhDYfgasaacH8akY=wiFfYdH8Gipec8Eeeu0xXdbba9frFj0=OqFfea0dXdd9vqai=hGuQ8kuc9pgc9s8qqaq=dirpe0xb9q8qiLsFr0=vr0=vr0dc8meaabaqaciaacaGaaeqabaqabeGadaaakeaacuWGsbGugaacaiabcIcaOiabdsha0jabcMcaPiabg2da9maabmaabaqbaeqabqabaaaaaeaacqWG4baEdaWgaaWcbaGaeGymaeJaeGymaedabeaakiabcIcaOiabdsha0jabcMcaPaqaaiabdIha4naaBaaaleaacqaIXaqmcqaIYaGmaeqaaOGaeiikaGIaemiDaqNaeiykaKcabaGaee4saSeabaGaemiEaG3aaSbaaSqaaiabigdaXiabd2gaTbqabaGccqGGOaakcqWG0baDcqGGPaqkaeaacqWG4baEdaWgaaWcbaGaeGOmaiJaeGymaedabeaakiabcIcaOiabdsha0jabcMcaPaqaaiabdIha4naaBaaaleaacqaIYaGmcqaIYaGmaeqaaOGaeiikaGIaemiDaqNaeiykaKcabaGaee4saSeabaGaemiEaG3aaSbaaSqaaiabikdaYiabd2gaTbqabaGccqGGOaakcqWG0baDcqGGPaqkaeaacqqGnbqtaeaacqqGnbqtaeaacqqGpbWtcqqGGaaicqWG4baEdaWgaaWcbaGaemyAaKMaemOAaOgabeaakiabcIcaOiabdsha0jabcMcaPaqaaiabb2eanbqaaiabdIha4naaBaaaleaacqWGUbGBcqaIXaqmaeqaaOGaeiikaGIaemiDaqNaeiykaKcabaGaemiEaG3aaSbaaSqaaiabd6gaUjabikdaYaqabaGccqGGOaakcqWG0baDcqGGPaqkaeaacqqGlbWsaeaacqqGpbWtcqqGGaaicqWG4baEdaWgaaWcbaGaemOBa4MaemyBa0gabeaakiabcIcaOiabdsha0jabcMcaPaaaaiaawIcacaGLPaaacaWLjaGaaCzcamaabmaabaGaeGymaedacaGLOaGaayzkaaaaaa@8899@

Each column represents a response vector associated with a particular odour, whereas the rows are the responses of an individual sensor to the different odours. As odour sensors do not in general behave independently, an individual sensor will typically respond to odours but with varying cross-sensitivity and intensity. As a result, the off-diagonal terms of *R *are usually nonzero, and thus, under these conditions, non-trivial data processing techniques (i.e. pattern analysis) are required to process the data and extract knowledge [1-4].

### 4.2 Feature extraction

There is certainly some distinguishing feature among the classes, which is unknown to us. So we want to take some of the known features and explore their capability of classification.

For each sample we know responses of 18 sensors over 120 seconds. The data from a sensor for each sample is a time-series data having 120 consecutive values. So we know *x*_*ij *_(*t*) for *t *= 1,..,120. We want to get just a scalar value in place of *x*_*ij *_(*t*) as a representative value. Basically the feature extraction task extracts an 18 × 1 array from 18 × 120 values. So we want to convert the time dependent matrix R˜
 MathType@MTEF@5@5@+=feaafiart1ev1aaatCvAUfKttLearuWrP9MDH5MBPbIqV92AaeXatLxBI9gBaebbnrfifHhDYfgasaacH8akY=wiFfYdH8Gipec8Eeeu0xXdbba9frFj0=OqFfea0dXdd9vqai=hGuQ8kuc9pgc9s8qqaq=dirpe0xb9q8qiLsFr0=vr0=vr0dc8meaabaqaciaacaGaaeqabaqabeGadaaakeaacuWGsbGugaacaaaa@2DE8@(*t*) to R˜
 MathType@MTEF@5@5@+=feaafiart1ev1aaatCvAUfKttLearuWrP9MDH5MBPbIqV92AaeXatLxBI9gBaebbnrfifHhDYfgasaacH8akY=wiFfYdH8Gipec8Eeeu0xXdbba9frFj0=OqFfea0dXdd9vqai=hGuQ8kuc9pgc9s8qqaq=dirpe0xb9q8qiLsFr0=vr0=vr0dc8meaabaqaciaacaGaaeqabaqabeGadaaakeaacuWGsbGugaacaaaa@2DE8@ which is independent of time. This will be our knowledge base. By plotting the 120 values of *x*_*ij *_(*t*) against time we get a graph. This graph approximates the function which determines the responses of the sensor over the time.

**Figure 1:**
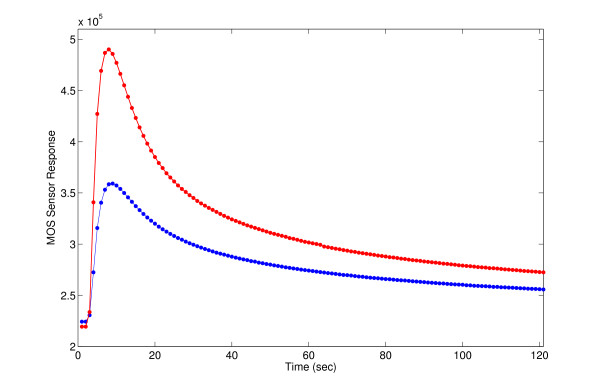
Sensor response curves from ENT bacteria sniffing experiments.

Figure 1 shows the characteristic feature and shape of the sensor response curves from our experiments.

There are different feature extraction techniques available for this type of response curve. The generally used "scalar feature extraction (*x*_*ij *_(*t*) transforms into a scalar quantity)" techniques are following ones. Let *x*_*ij *_(*t*) be the measured signal of the *i-th *sensor of *j-th *odour. If it is a steady-state signal, the custom is to choose the feature as the difference between the steady-state response and the base-line response. For transient signals, number of features is richer. The most popular feature is, again, the difference between the signal's peak and its baseline. Other options are to take the area beneath the curve; the area beneath the curve left of the peak, and the time it takes for the signal to reach its peak.

One of the commonest "vector feature extraction (*x*_*ij *_(*t*) transforms into a time independent vector quantity)" technique is the wavelet analysis of the data available. A lot of work has been done by Discante *et. al *[12,13]. They tried different dictionary of functions to optimize the number of scalar entities in the extracted feature vector. But they successfully optimized the number to 5, compromising 10% classification error. In other approaches the response functions have been modeled with some known models and their estimated parameters were used as features. But in that case also "vector feature extraction" worked better than "scalar feature extraction" [14].

**Figure 2:**
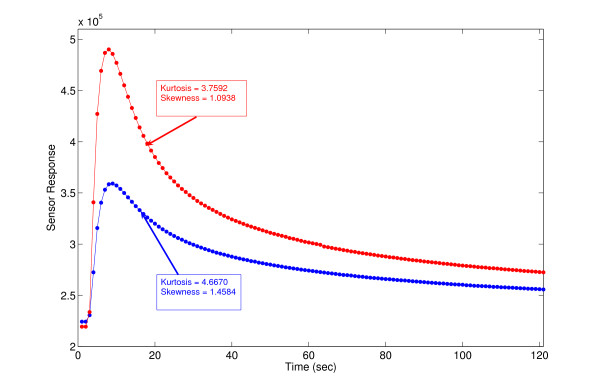
Example for calculated Skewness and Kurtosis (discussed in appendix) of the sensory curve as new type of extracted feature for knowledge representation.

In historical time, to minimize the complexity and time of calculation, it has been tried to minimize the no. of entries in vector of the features. So the optimum case is the scalar feature. Here we have studied a set of new scalar features, not used before. They are *skewness *and *kurtosis *(discussed in appendix) of the data. These features are excellent discriminator between different types of steady-state response curves. In the following Figure 2, we have some examples of calculated values of *skewness *and *kurtosis *features. We compared these two newly extracted features with four of the well-known scalar features (namely, difference between signal's peak and its baseline, area beneath the signal curve, area beneath the signal curve (left of the peak), required time for the signal to reach the peak) in the later section of this paper.

These two features are well known in cases of checking symmetry of the curves. But here they are giving some measure of the asymmetry of the asymmetric curves. The patterns of asymmetry judged by them, speaks for the various things like, peak of the stimulus, rate of the stimulus going up and falling down or in some cases their time of action in precise.

In many approaches, more than one feature per signal is used, working with a subset of the aforementioned features. So these two new scalar features can be an addition to that super set. These features can lead us to a new exhaustive set of features.

## 5. Multi-featured knowledge representation

To represent knowledge from the experimental data, generation of patterns and study of pattern recognition algorithms is necessary. Pattern recognition is defined as the process of identifying structure in data by comparison to known structure. Patterns are typically described in terms of multidimensional data vectors, where each component is called a feature. The aim of a pattern recognition system is to associate each pattern with one of the possible pattern classes (or simply classes). Obviously, different patterns should be associated with the same class or with different classes depending on whether they are characterized by similar or dissimilar features, respectively. In the case of the e-nose, the patterns and the classes are, respectively, the responses of the sensor array to odorants, and the odorants being considered [15-19].

After the data representation stage the next step is knowledge representation from the pre-processed data.

After individual feature extraction, each sample is being represented by an 18 × 1 valued array i.e., the data gathered from a sample. If in the problem we have k classes, then in the knowledge we will have *N*_*g *_samples from g-th class when(*g *= 1,....., *k*), and sample data will be gathered from them by the sensors. So after feature extraction g-th class contains *N*_*g *_arrays of dimension 18 × 1. This collection of data is known as knowledge.

In this study we have extracted six different types of features from our raw sensory data. So the overall knowledge representation for these ENT bacteria classification study was based on six multiple features:

1. Difference between signal's peak and its baseline

2. Area beneath the signal curve

3. Area beneath the signal curve (left of the peak)

4. Required time for the signal to reach the peak

5. Skewness

6. Kurtosis

This multi-featured knowledge base can be used to train an artificial neural network (ANN) for supervised bacteria classification or this featured based knowledgebase can be used to develop a conventional fuzzy rule based system for unsupervised unknown sample classification.

When a new sample will be examined, we will extract feature from that in the same manner as the knowledge has been generated. Then this knowledge will be used to classify this new unknown data by using following classification techniques.

## 6. The problem being solved

Rapid screening and diagnosis of ENT infection in hospital environment is a challenging problem to solve. There is a basic need in all hospitals for novel technique for rapid bacteria screening. This is required because present conventional pathological lab based method is,

1. Expensive

2. Lengthy Time consuming Process

3. More use of infrastructure which increase operating cost

4. Less Care as results are always delayed

Typical time required to get a single bacteria screening result pathological lab is 24 – 72 hrs. This problem is so serious that every year NHS spends £1 B just to tackle only MRSA super bug. But still the overall success rate of proper diagnosis of ENT infection in hospital environment in right time is only 65%. So it's an ongoing serious problem and virtually impossible to solve with the existing method [1,7,8,25,26]. Researchers have started looking at different alternative methods to find a novel rapid screening and diagnosis of ENT infections for last 5 years. In our previous research papers we have looked in "Intelligent Adaptive Sensory Signal Processing (IASSP)" techniques for solving this persistent problem in hospital environment. It has been proved from several research papers that E-nose based on IASSP methods could be a very effective, novel and rapid solution for screening and diagnosis of ENT infections.

IASSP is the heart of the gas sensor based 'Electronic Nose' concept. All intelligence of the E-nose system is in the IASSP blocks combined with it.

E-nose based on IASSP method takes 5–15 min to give the result for single bacteria screening, which is,

1. Very Rapid diagnosis

2. Less Expensive

3. Less use of infrastructure and operating cost

4. Better Care

### 6.1 *PCA *for data estimation

PCA is a linear supervised method that has been widely used by various researchers to discriminate the response of an e-nose to simple and complex odours [20-24]. It is a multivariate statistical method, based on the Karhunen-Lowve expansion, used in classification models to produce classification results for e-nose pattern recognition techniques. The method consists of expressing the response vectors *r*_*ij *_in terms of linear combinations of orthogonal vectors, and is sometimes referred to as vector decomposition. Each orthogonal vector, principal component (PC), accounts for a certain amount of variance in the data with a decreasing degree of importance. The scalar product of the orthogonal vectors with the response vector gives the value of the *p*th principal components:

*X*_*p *_= *α*_1*p*_*r*_1*j *_+ *α*_2*p*_*r*_2*j *_+ ... + *α*_*ip*_*r*_*ij *_+ ... + *α*_*np*_*r*_*nj *_    (2)

The variance of each PC score, *X*_*p*_, is maximized under the constraint that the sum of the coefficients of the orthogonal vectors or eigenvectors *α*_*p *_= (*α*_*1p*_... *α*_*jp*_... *α*_*np*_) is set to unity, and the vectors are uncorrelated. Since there is often a high degree of sensor co-linearity in e-nose data, the majority of the information held in response space can often be displayed using a small number of PCs. PCA is in essence a data reduction technique for correlated data, such that a *n*-dimensional problem can be described by a two or three dimensional plot. It can be applied to high dimensional data-sets to identify their variation in structure for discrimination in gas sensor applications.

**Figure 3:**
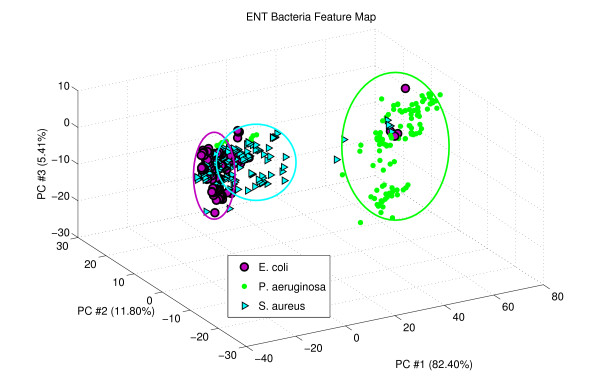
PCA component plot of the data using "area under the curve" as feature.

We have done PC analysis using "area beneath the curve", "kurtosis" and "skewness" as feature and plotted first three significant PCA components to estimate characteristics of the data. Feature "area beneath the curve" based PCA results indicated that the data from different classes are closely overlapped in some regions and they do have a reasonable amount of outliers to think about them. See figure 3 where specifically S. aureus PCA scores are overlapped heavily with E. coli PCA scores. Microbiologically it's true that E. coli and S. aureus strains should have similar characteristics (they can live together) and that feature is also reflected in our initial PCA results. The overlapping clusters have significant effect on over all classification and it also increases the rate of misclassifications.

**Figure 4:**
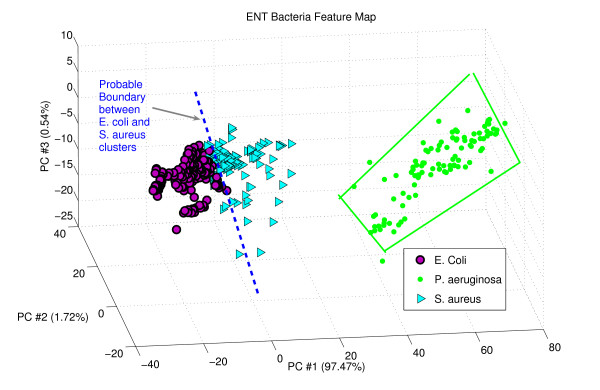
PCA component plot of the data using "skewness" as feature.

In the next stage of PCA based on features like "kurtosis" and "skewness" indicate a better picture for us. See figure 4 where specifically S. aureus PCA scores are overlapped with E. coli PCA scores at some points but not like in figure 3. We can very easily estimate a probable linear boundary in between these two clusters for classification. From this PCA results we can conclude that rate of misclassification will much less if we consider "kurtosis" and "skewness" as principle feature to develop our ENT bacteria knowledgebase. This indicates the effectiveness of the new feature "kurtosis" and "skewness". It is also important to note that in figure 4 percentage of information variation along PC#1 (97.47%) is higher than percentage of information variation along PC#1 (82.4%) in Figure 3. It is proved that from this estimation study that features like "kurtosis" and "skewness" could help to give a much better classification rate.

P. aeruginosa cluster is very clear in both of the figures 3 and 4. Although there are some overlapping in figure 3, but in the figure 4 P. aeruginosa cluster is very clear with no overlapping.

### 6.2 PCA for sub-classification of S. aureus strains

Our early work has proved that it is possible to classify MRSA and MSSA strains on the basis of headspace analysis by using an intelligent conducting polymer sensor based e-nose system. Now it is an important challenge to prove the S. aureus classification is possible from E. coli and P. aeruginosa, and in the next stage sub-classification of the original S. aureus data into two sub-clusters for MRSA and MSSA is also possible.

We have done PC analysis using "kurtosis" and "skewness" as feature and plotted first three significant PCA components to estimate inner characteristics of the S. aureus data cluster. Microbiological strain similarity between E. coli and S. aureus has a significant impact on the sub-classification of S. aureus strains into MRSA and MSSA, as some E. coli PC score point are very similar with some score points of MRSA and MSSA. But apart from those few point, there was now significant overlapping between them.

**Figure 5:**
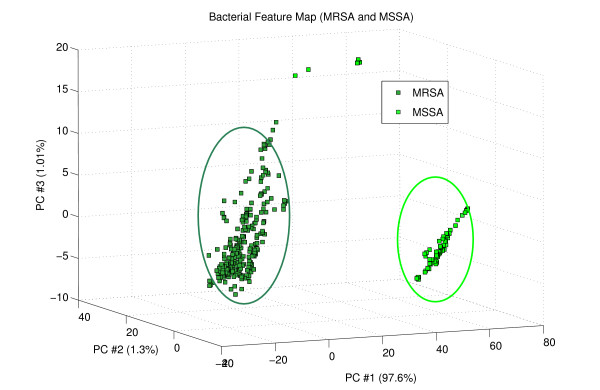
PCA component plot of the MRSA-MSSA data using "skewness" as features.

In figure 5 we have 97.6% of the total information variation along PC#1 for the MRSA-MSSA data using "kurtosis" and "skewness" as features. This estimation proves that new feature extraction technique can increase the linearity of the classification problem and also reduce the non-linearity. Also no overlap between these two clusters indicates that knowledge representation has been significantly improved by using "kurtosis" and "skewness" as features. This achievement will help to classify the unknown sample with high accuracy.

## 7. Classification methodology

### 7.1 Artificial Neural Networks approach

The six different bacteria dataset were analyzed using three supervised ANN classifiers, namely the Multi Layer Perceptron (MLP), Probabilistic Neural network (PNN) and Radial basis function network (RBF) paradigms [2-4,27]. Training of the neural networks was performed with 80% of the whole data set. The remaining 20% of the whole data were used for testing the neural networks. These percentages were selected arbitrarily and were applied for all data sets. The aim of this comparative study was to identify the most appropriate ANN paradigm, which can be trained with best accuracy, to predict the "type of ENT infections" or in other words "type of ENT bacteria".

#### 7.1.1 MLP classifier

**Figure 6:**
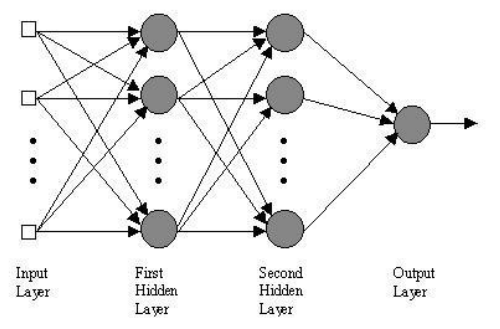
A graphical representation of an MLP.

The most common neural network model is the multilayer perceptron (MLP). This type of neural network is known as a supervised network because it requires a desired output in order to learn. The goal of this type of network is to create a model that correctly maps the input to the output using historical data so that the model can then be used to produce the output when the desired output is unknown. A graphical representation of an MLP is shown below in Figure 6.

#### 7.1.2 RBF classifier

These networks have a static Gaussian function as the nonlinearity for the hidden layer processing elements. The Gaussian function responds only to a small region of the input space around the Gaussian centered. The key to a successful implementation of these networks is to find suitable centres for the Gaussian functions. This can be done with supervised learning, but an unsupervised approach usually produces better results. For this reason, NeuroSolutions implements RBF networks as a hybrid supervised-unsupervised topology.

The simulation starts with the training of an unsupervised layer. Its function is to derive the Gaussian centres and the widths from the input data. These centres are encoded within the weights of the unsupervised layer using competitive learning. During the unsupervised learning, the widths of the Gaussians are computed based on the centres of their neighbors. The output of this layer is derived from the input data weighted by a Gaussian mixture.

Once the unsupervised layer has completed its training, the supervised segment then sets the centres of Gaussian functions (based on the weights of the unsupervised layer) and determines the width (standard deviation) of each Gaussian. Any supervised topology (such as a MLP) may be used for the classification of the weighted input.

**Figure 7:**
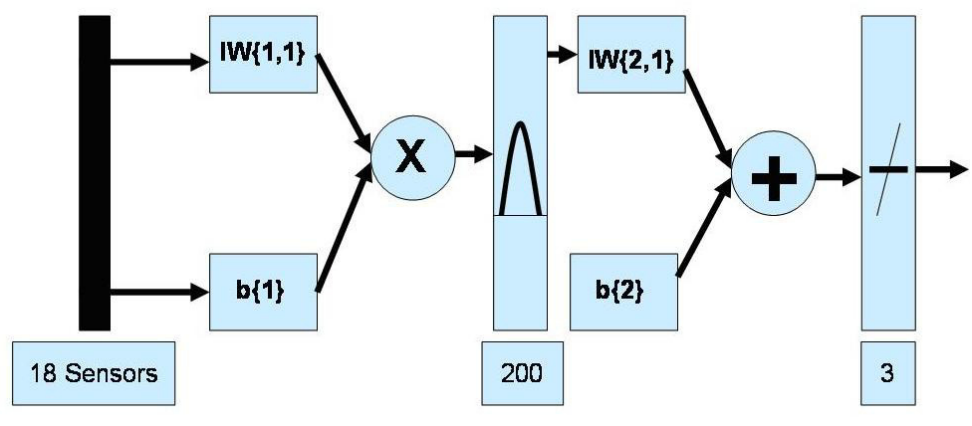
A graphical representation of an RBF Network used.

The advantage of the radial basis function network is that it finds the input to output map using local approximators. Usually the supervised segment is simply a linear combination of the approximators. Since linear combiners have few weights, these networks train extremely fast and require fewer training samples. A graphical representation of an RBF is shown below in Figure 7.

#### 7.1.3 PNN classifier

The PNN networks are variants of the radial basis function (RBF) network. Unlike the standard RBF, the weights of theses networks can be calculated analytically. In this case, the number of cluster centers is by definition equal to the number of exemplars, and they are all set to the same variance.

### 7.2 "Maximum Probability Rule" based classification

The selection of a patient is done randomly from a large collection of patients having a specific disease, so that the collected samples can be treated as randomly drawn samples. Here we denote each sample as a unit "u", reflecting one specific case of disease or in other word one specific class of bacteria. Now the feature extracted from the sensor response is treated as the observation vector X_*u *_of unit u. In our knowledge for each class we have some observation vectors (of dimension 18 × 1) of randomly selected sample units from that class. We denoted the number of units in a class in the knowledge by N_*g *_when g represents that specific class [1,28,29].

#### 7.2.1 Basic idea: modeling of each class by a probability model

At the preliminary stage, we assign probability models to each of the classes. We assume that if we draw random samples from one class it will be selected from a fixed probability distribution, which is specific for that class. In general this means that the bacteria class has been modeled with that probability distribution model. So we assign a distribution to each of the classes. Assuming continuous probability models we specify a probability density function f(X/g) for class g.

#### 7.2.3 Decision rule: maximum (Bayesian) probability rule

**Baye's Rule**: The posterior and prior probabilities of class membership are related using typicality probability of class by Baye's Rule in the following manner,

P(g/Xu)=πg⋅P(Xu/g)∑g′=1kπg′⋅P(Xu/g′)     (3)
 MathType@MTEF@5@5@+=feaafiart1ev1aaatCvAUfKttLearuWrP9MDH5MBPbIqV92AaeXatLxBI9gBaebbnrfifHhDYfgasaacH8akY=wiFfYdH8Gipec8Eeeu0xXdbba9frFj0=OqFfea0dXdd9vqai=hGuQ8kuc9pgc9s8qqaq=dirpe0xb9q8qiLsFr0=vr0=vr0dc8meaabaqaciaacaGaaeqabaqabeGadaaakeaacqWGqbaucqGGOaakcqWGNbWzcqGGVaWlcqqGybawdaWgaaWcbaGaeeyDauhabeaakiabcMcaPiabg2da9maalaaabaacciGae8hWda3aaSbaaSqaaiabdEgaNbqabaGccqGHflY1cqWGqbaucqGGOaakcqqGybawdaWgaaWcbaGaeeyDauhabeaakiabc+caViabdEgaNjabcMcaPaqaamaaqahabaGae8hWda3aaSbaaSqaaiqbdEgaNzaafaaabeaaaeaacuWGNbWzgaqbaiabg2da9iabigdaXaqaaiabdUgaRbqdcqGHris5aOGaeyyXICTaemiuaaLaeiikaGIaeeiwaG1aaSbaaSqaaiabbwha1bqabaGccqGGVaWlcuWGNbWzgaqbaiabcMcaPaaacaWLjaGaaCzcamaabmaabaGaeG4mamdacaGLOaGaayzkaaaaaa@5B96@

So for classification purpose of a new unit of observation u to any one of the classes, our **decision rule **becomes:

**Assign unit u to class g if P(g/X_*u*_) > P(h/X_*u*_) for g ≠ h**.

This is called the "**Maximum Probability Rule**".

Again k values of P(X_*u*_/g) need to be determined for each unit. Because the denominator in 1 is constant for all class, the rule could more simply be based on the k values of *π*_*g*_·f(X_*u*_/g) and 1 can be stated equivalently as,

P(g/Xu)=πg⋅f(Xu/g)∑g′=1kπg′⋅f(Xu/g′)     (4)
 MathType@MTEF@5@5@+=feaafiart1ev1aaatCvAUfKttLearuWrP9MDH5MBPbIqV92AaeXatLxBI9gBaebbnrfifHhDYfgasaacH8akY=wiFfYdH8Gipec8Eeeu0xXdbba9frFj0=OqFfea0dXdd9vqai=hGuQ8kuc9pgc9s8qqaq=dirpe0xb9q8qiLsFr0=vr0=vr0dc8meaabaqaciaacaGaaeqabaqabeGadaaakeaacqWGqbaucqGGOaakcqWGNbWzcqGGVaWlcqqGybawdaWgaaWcbaGaeeyDauhabeaakiabcMcaPiabg2da9maalaaabaacciGae8hWda3aaSbaaSqaaiabdEgaNbqabaGccqGHflY1cqWGMbGzcqGGOaakcqqGybawdaWgaaWcbaGaeeyDauhabeaakiabc+caViabdEgaNjabcMcaPaqaamaaqahabaGae8hWda3aaSbaaSqaaiqbdEgaNzaafaaabeaaaeaacuWGNbWzgaqbaiabg2da9iabigdaXaqaaiabdUgaRbqdcqGHris5aOGaeyyXICTaemOzayMaeiikaGIaeeiwaG1aaSbaaSqaaiabbwha1bqabaGccqGGVaWlcuWGNbWzgaqbaiabcMcaPaaacaWLjaGaaCzcamaabmaabaGaeGinaqdacaGLOaGaayzkaaaaaa@5BF0@

#### 7.2.4 Assignment of probability model

The "Maximum Probability Rule" involving posterior probability of class membership can be applied only if the probability density functions of the distributions of the classes are known [1,29].

Each class may be modeled by assigning a density function f(X/g). Our only knowledge about class g is the N_*g *_observation units of randomly drawn samples from class g. There are many commonly used techniques of estimating f(X/g) from this knowledge. Commonly these methods are divided in two classes, parametric and non-parametric ones.

Parametric approach: Specify a theoretical probability distributional model fθ˜
 MathType@MTEF@5@5@+=feaafiart1ev1aaatCvAUfKttLearuWrP9MDH5MBPbIqV92AaeXatLxBI9gBaebbnrfifHhDYfgasaacH8akY=wiFfYdH8Gipec8Eeeu0xXdbba9frFj0=OqFfea0dXdd9vqai=hGuQ8kuc9pgc9s8qqaq=dirpe0xb9q8qiLsFr0=vr0=vr0dc8meaabaqaciaacaGaaeqabaqabeGadaaakeaacqqGMbGzdaWgaaWcbaacciGaf8hUdeNba4baaeqaaaaa@300D@(X/g), assume that the data on hand fit the model and estimate the model parameters θ˜
 MathType@MTEF@5@5@+=feaafiart1ev1aaatCvAUfKttLearuWrP9MDH5MBPbIqV92AaeXatLxBI9gBaebbnrfifHhDYfgasaacH8akY=wiFfYdH8Gipec8Eeeu0xXdbba9frFj0=OqFfea0dXdd9vqai=hGuQ8kuc9pgc9s8qqaq=dirpe0xb9q8qiLsFr0=vr0=vr0dc8meaabaqaciaacaGaaeqabaqabeGadaaakeaaiiGacuWF4oqCgaGhaaaa@2E8E@ using the data, and construct a rule using these estimates.

Non-Parametric approach: Estimate the density values directly from data with no prior model specification, and construct a rule using these estimates.

We applied two significant methods from these two classes for classification of our ENT data. In parametric model multi-nominal model has been used, where in non-parametric approaches our choice was for "kernel density estimator". There are some other very successful non-parametric methods, like k-NN classification rule. It is true that the k-nn method is faster than kernel method. But where the minimization of classification error is also important then these two methods are equivalent when number of sensors is too large.

### 7.3 Non-parametric approach

In this approach we do not assume any pre-specified theoretical parametric form of **pdf **of a distribution. So it is also known as distribution-free method. There are four major types of non-parametric **pdf **estimators: the histogram, the kernel method, the k-nearest-neighbour method, and the series method. But in this case we will only use the kernel estimates of the pdf and a further development of this method, generally portrayed as adaptive kernel estimators.

#### 7.3.1 General kernel estimator based on Bayesian rule

In general the form of the kernel estimator is,

f^(Xu/g)=1Ng∑i=1NgK(Xu−Xi)     (5)
 MathType@MTEF@5@5@+=feaafiart1ev1aaatCvAUfKttLearuWrP9MDH5MBPbIqV92AaeXatLxBI9gBaebbnrfifHhDYfgasaacH8akY=wiFfYdH8Gipec8Eeeu0xXdbba9frFj0=OqFfea0dXdd9vqai=hGuQ8kuc9pgc9s8qqaq=dirpe0xb9q8qiLsFr0=vr0=vr0dc8meaabaqaciaacaGaaeqabaqabeGadaaakeaacuWGMbGzgaqcaiabcIcaOiabdIfaynaaBaaaleaacqWG1bqDaeqaaOGaei4la8Iaem4zaCMaeiykaKIaeyypa0ZaaSaaaeaacqaIXaqmaeaacqWGobGtdaWgaaWcbaGaem4zaCgabeaaaaGcdaaeWbqaaiabdUealjabcIcaOiabdIfaynaaBaaaleaacqWG1bqDaeqaaOGaeyOeI0IaemiwaG1aaSbaaSqaaiabdMgaPbqabaGccqGGPaqkaSqaaiabdMgaPjabg2da9iabigdaXaqaaiabd6eaonaaBaaameaacqWGNbWzaeqaaaqdcqGHris5aOGaaCzcaiaaxMaadaqadaqaaiabiwda1aGaayjkaiaawMcaaaaa@4F0F@

Imposing the conditions *K*(*z*) ≥ 0 and ∫*K*(*z*)*dz *= 1 on K, then it is easy to see that f^
 MathType@MTEF@5@5@+=feaafiart1ev1aaatCvAUfKttLearuWrP9MDH5MBPbIqV92AaeXatLxBI9gBaebbnrfifHhDYfgasaacH8akY=wiFfYdH8Gipec8Eeeu0xXdbba9frFj0=OqFfea0dXdd9vqai=hGuQ8kuc9pgc9s8qqaq=dirpe0xb9q8qiLsFr0=vr0=vr0dc8meaabaqaciaacaGaaeqabaqabeGadaaakeaacuWGMbGzgaqcaaaa@2E11@ also satisfies f^
 MathType@MTEF@5@5@+=feaafiart1ev1aaatCvAUfKttLearuWrP9MDH5MBPbIqV92AaeXatLxBI9gBaebbnrfifHhDYfgasaacH8akY=wiFfYdH8Gipec8Eeeu0xXdbba9frFj0=OqFfea0dXdd9vqai=hGuQ8kuc9pgc9s8qqaq=dirpe0xb9q8qiLsFr0=vr0=vr0dc8meaabaqaciaacaGaaeqabaqabeGadaaakeaacuWGMbGzgaqcaaaa@2E11@(*z*) ≥ 0 and ∫f^
 MathType@MTEF@5@5@+=feaafiart1ev1aaatCvAUfKttLearuWrP9MDH5MBPbIqV92AaeXatLxBI9gBaebbnrfifHhDYfgasaacH8akY=wiFfYdH8Gipec8Eeeu0xXdbba9frFj0=OqFfea0dXdd9vqai=hGuQ8kuc9pgc9s8qqaq=dirpe0xb9q8qiLsFr0=vr0=vr0dc8meaabaqaciaacaGaaeqabaqabeGadaaakeaacuWGMbGzgaqcaaaa@2E11@(*z*)*dz *= 1 so that f^
 MathType@MTEF@5@5@+=feaafiart1ev1aaatCvAUfKttLearuWrP9MDH5MBPbIqV92AaeXatLxBI9gBaebbnrfifHhDYfgasaacH8akY=wiFfYdH8Gipec8Eeeu0xXdbba9frFj0=OqFfea0dXdd9vqai=hGuQ8kuc9pgc9s8qqaq=dirpe0xb9q8qiLsFr0=vr0=vr0dc8meaabaqaciaacaGaaeqabaqabeGadaaakeaacuWGMbGzgaqcaaaa@2E11@ is a legitimate pdf. Using product form of the kernel estimates we get,

f^(Xu/g)=1Ng∑i=1Ng∏j=1p1hjgK(Xuj−Xijhjg)     (6)
 MathType@MTEF@5@5@+=feaafiart1ev1aaatCvAUfKttLearuWrP9MDH5MBPbIqV92AaeXatLxBI9gBaebbnrfifHhDYfgasaacH8akY=wiFfYdH8Gipec8Eeeu0xXdbba9frFj0=OqFfea0dXdd9vqai=hGuQ8kuc9pgc9s8qqaq=dirpe0xb9q8qiLsFr0=vr0=vr0dc8meaabaqaciaacaGaaeqabaqabeGadaaakeaacuWGMbGzgaqcaiabcIcaOiabdIfaynaaBaaaleaacqWG1bqDaeqaaOGaei4la8Iaem4zaCMaeiykaKIaeyypa0ZaaSaaaeaacqaIXaqmaeaacqWGobGtdaWgaaWcbaGaem4zaCgabeaaaaGcdaaeWbqaamaarahabaWaaSaaaeaacqaIXaqmaeaacqWGObaAdaWgaaWcbaGaemOAaOMaem4zaCgabeaaaaGccqWGlbWscqGGOaakdaWcaaqaaiabdIfaynaaBaaaleaacqWG1bqDcqWGQbGAaeqaaOGaeyOeI0IaemiwaG1aaSbaaSqaaiabdMgaPjabdQgaQbqabaaakeaacqWGObaAdaWgaaWcbaGaemOAaOMaem4zaCgabeaaaaGccqGGPaqkaSqaaiabdQgaQjabg2da9iabigdaXaqaaiabdchaWbqdcqGHpis1aaWcbaGaemyAaKMaeyypa0JaeGymaedabaGaemOta40aaSbaaWqaaiabdEgaNbqabaaaniabggHiLdGccaWLjaGaaCzcamaabmaabaGaeGOnaydacaGLOaGaayzkaaaaaa@624E@

Here the K is known as kernel function and {hjg}j=1,p
 MathType@MTEF@5@5@+=feaafiart1ev1aaatCvAUfKttLearuWrP9MDH5MBPbIqV92AaeXatLxBI9gBaebbnrfifHhDYfgasaacH8akY=wiFfYdH8Gipec8Eeeu0xXdbba9frFj0=OqFfea0dXdd9vqai=hGuQ8kuc9pgc9s8qqaq=dirpe0xb9q8qiLsFr0=vr0=vr0dc8meaabaqaciaacaGaaeqabaqabeGadaaakeaadaGadeqaaiabdIgaOnaaBaaaleaacqWGQbGAcqWGNbWzaeqaaaGccaGL7bGaayzFaaWaaSbaaSqaaiabdQgaQjabg2da9iabigdaXiabcYcaSiabdchaWbqabaaaaa@38E9@ is called the smoothing parameters for the g-th class.

In our practice we use the **normal kernel functions **and take equal values for all {hjg}j=1,p
 MathType@MTEF@5@5@+=feaafiart1ev1aaatCvAUfKttLearuWrP9MDH5MBPbIqV92AaeXatLxBI9gBaebbnrfifHhDYfgasaacH8akY=wiFfYdH8Gipec8Eeeu0xXdbba9frFj0=OqFfea0dXdd9vqai=hGuQ8kuc9pgc9s8qqaq=dirpe0xb9q8qiLsFr0=vr0=vr0dc8meaabaqaciaacaGaaeqabaqabeGadaaakeaadaGadeqaaiabdIgaOnaaBaaaleaacqWGQbGAcqWGNbWzaeqaaaGccaGL7bGaayzFaaWaaSbaaSqaaiabdQgaQjabg2da9iabigdaXiabcYcaSiabdchaWbqabaaaaa@38E9@ of g-th class, then our estimator becomes

f^(Xu/g)=1Ng∑i=1Ng∏j=1p1hgexp⁡[−12(Xuj−Xijhg)]     (7)
 MathType@MTEF@5@5@+=feaafiart1ev1aaatCvAUfKttLearuWrP9MDH5MBPbIqV92AaeXatLxBI9gBaebbnrfifHhDYfgasaacH8akY=wiFfYdH8Gipec8Eeeu0xXdbba9frFj0=OqFfea0dXdd9vqai=hGuQ8kuc9pgc9s8qqaq=dirpe0xb9q8qiLsFr0=vr0=vr0dc8meaabaqaciaacaGaaeqabaqabeGadaaakeaacuWGMbGzgaqcaiabcIcaOiabdIfaynaaBaaaleaacqWG1bqDaeqaaOGaei4la8Iaem4zaCMaeiykaKIaeyypa0ZaaSaaaeaacqaIXaqmaeaacqWGobGtdaWgaaWcbaGaem4zaCgabeaaaaGcdaaeWbqaamaarahabaWaaSaaaeaacqaIXaqmaeaacqWGObaAdaWgaaWcbaGaem4zaCgabeaaaaaabaGaemOAaOMaeyypa0JaeGymaedabaGaemiCaahaniabg+GivdaaleaacqWGPbqAcqGH9aqpcqaIXaqmaeaacqWGobGtdaWgaaadbaGaem4zaCgabeaaa0GaeyyeIuoakiGbcwgaLjabcIha4jabcchaWjabcUfaBjabgkHiTmaalaaabaGaeGymaedabaGaeGOmaidaaiabcIcaOmaalaaabaGaemiwaG1aaSbaaSqaaiabdwha1jabdQgaQbqabaGccqGHsislcqWGybawdaWgaaWcbaGaemyAaKMaemOAaOgabeaaaOqaaiabdIgaOnaaBaaaleaacqWGNbWzaeqaaaaakiabcMcaPiabc2faDjaaxMaacaWLjaWaaeWaaeaacqaI3aWnaiaawIcacaGLPaaaaaa@67F5@

Here we estimated h values for each class g and denoted them as *h*_*g*_.

#### 7.3.2 Adaptive kernel estimator as IBC

A practical drawback of the kernel method of density estimation is its inability to deal satisfactorily with the tails of distributions without over smoothing the main part of the density. The data have reasonable amount of outliers and their densities are multimodal densities. So the general method smoothed the inner part of the density, where it should not be due to the close overlapping of the data. This pattern of the data speaks for a density estimator which can sense small masses of probability and also a robust one at the same time. From the kernel density point of view, if the window function can be adopted locally depending upon the local data, we can have the optimum one. So here we used one of the popularly known adaptive approaches, adaptive kernel method. In this method another added local smoothing parameter has been used with the global smoothing parameter. This local one has been estimated from a pilot estimate of the density. Previous works show that this method tackles the tail probabilities excellently [1,28-30].

The adaptive kernel estimators will be,

f^(Xu/g)=1Ng∑i=1Ng1hig−pK(Xu−Xi)     (8)
 MathType@MTEF@5@5@+=feaafiart1ev1aaatCvAUfKttLearuWrP9MDH5MBPbIqV92AaeXatLxBI9gBaebbnrfifHhDYfgasaacH8akY=wiFfYdH8Gipec8Eeeu0xXdbba9frFj0=OqFfea0dXdd9vqai=hGuQ8kuc9pgc9s8qqaq=dirpe0xb9q8qiLsFr0=vr0=vr0dc8meaabaqaciaacaGaaeqabaqabeGadaaakeaacuWGMbGzgaqcaiabcIcaOiabdIfaynaaBaaaleaacqWG1bqDaeqaaOGaei4la8Iaem4zaCMaeiykaKIaeyypa0ZaaSaaaeaacqaIXaqmaeaacqWGobGtdaWgaaWcbaGaem4zaCgabeaaaaGcdaaeWbqaamaalaaabaGaeGymaedabaGaemiAaG2aa0baaSqaaiabdMgaPjabdEgaNbqaaiabgkHiTiabdchaWbaaaaaabaGaemyAaKMaeyypa0JaeGymaedabaGaemOta40aaSbaaWqaaiabdEgaNbqabaaaniabggHiLdGccqWGlbWscqGGOaakcqWGybawdaWgaaWcbaGaemyDauhabeaakiabgkHiTiabdIfaynaaBaaaleaacqWGPbqAaeqaaOGaeiykaKIaaCzcaiaaxMaadaqadaqaaiabiIda4aGaayjkaiaawMcaaaaa@5698@

where the *N*_*g *_smoothing parameters *h*_*ig *_(*i *= 1,......., *N*_*g*_) are based on some pilot estimate of the density f˜
 MathType@MTEF@5@5@+=feaafiart1ev1aaatCvAUfKttLearuWrP9MDH5MBPbIqV92AaeXatLxBI9gBaebbnrfifHhDYfgasaacH8akY=wiFfYdH8Gipec8Eeeu0xXdbba9frFj0=OqFfea0dXdd9vqai=hGuQ8kuc9pgc9s8qqaq=dirpe0xb9q8qiLsFr0=vr0=vr0dc8meaabaqaciaacaGaaeqabaqabeGadaaakeaacuWGMbGzgaacaaaa@2E10@(*X*/*g*). The smoothing parameters *h*_*ig *_are specified as *h*_*g *_*a*_*ig*_, where *h*_*g *_is a global smoothing parameter of a class and *a*_*ig *_are local smoothing parameters given by

aig={f˜(Xig/g)/Cg}−αg(i=1,.........,Ng)     (9)
 MathType@MTEF@5@5@+=feaafiart1ev1aaatCvAUfKttLearuWrP9MDH5MBPbIqV92AaeXatLxBI9gBaebbnrfifHhDYfgasaacH8akY=wiFfYdH8Gipec8Eeeu0xXdbba9frFj0=OqFfea0dXdd9vqai=hGuQ8kuc9pgc9s8qqaq=dirpe0xb9q8qiLsFr0=vr0=vr0dc8meaabaqaciaacaGaaeqabaqabeGadaaakeaafaqabeqacaaabaGaemyyae2aaSbaaSqaaiabdMgaPjabdEgaNbqabaGccqGH9aqpdaGadeqaamaalyaabaGafmOzayMbaGaacqGGOaakcqWGybawdaWgaaWcbaGaemyAaKMaem4zaCgabeaakiabc+caViabdEgaNjabcMcaPaqaaiabdoeadnaaBaaaleaacqWGNbWzaeqaaaaaaOGaay5Eaiaaw2haamaaCaaaleqabaGaeyOeI0ccciGae8xSde2aaSbaaWqaaiabdEgaNbqabaaaaaGcbaGaeiikaGIaemyAaKMaeyypa0JaeGymaeJaeiilaWIaeiOla4IaeiOla4IaeiOla4IaeiOla4IaeiOla4IaeiOla4IaeiOla4IaeiOla4IaeiOla4IaeiilaWIaemOta40aaSbaaSqaaiabdEgaNbqabaGccqGGPaqkaaGaaCzcaiaaxMaadaqadaqaaiabiMda5aGaayjkaiaawMcaaaaa@59DE@

where log⁡Cg=1Ng∑i=1Nglog⁡(f˜(Xig/g))
 MathType@MTEF@5@5@+=feaafiart1ev1aaatCvAUfKttLearuWrP9MDH5MBPbIqV92AaeXatLxBI9gBaebbnrfifHhDYfgasaacH8akY=wiFfYdH8Gipec8Eeeu0xXdbba9frFj0=OqFfea0dXdd9vqai=hGuQ8kuc9pgc9s8qqaq=dirpe0xb9q8qiLsFr0=vr0=vr0dc8meaabaqaciaacaGaaeqabaqabeGadaaakeaacyGGSbaBcqGGVbWBcqGGNbWzcqWGdbWqdaWgaaWcbaGaem4zaCgabeaakiabg2da9maalaaabaGaeGymaedabaGaemOta40aaSbaaSqaaiabdEgaNbqabaaaaOWaaabCaeaacyGGSbaBcqGGVbWBcqGGNbWzcqGGOaakcuWGMbGzgaacaiabcIcaOiabdIfaynaaBaaaleaacqWGPbqAcqWGNbWzaeqaaOGaei4la8Iaem4zaCMaeiykaKIaeiykaKcaleaacqWGPbqAcqGH9aqpcqaIXaqmaeaacqWGobGtdaWgaaadbaGaem4zaCgabeaaa0GaeyyeIuoaaaa@4F9E@

and f˜
 MathType@MTEF@5@5@+=feaafiart1ev1aaatCvAUfKttLearuWrP9MDH5MBPbIqV92AaeXatLxBI9gBaebbnrfifHhDYfgasaacH8akY=wiFfYdH8Gipec8Eeeu0xXdbba9frFj0=OqFfea0dXdd9vqai=hGuQ8kuc9pgc9s8qqaq=dirpe0xb9q8qiLsFr0=vr0=vr0dc8meaabaqaciaacaGaaeqabaqabeGadaaakeaacuWGMbGzgaacaaaa@2E10@(*X*_*ig*_/*g*) > 0 (*i *= 1,........, *N*_*g*_), and where *α*_*g *_is the sensitivity parameter satisfying 0 ≤ *α*_*g *_≤ 1. In practical application we assumed *α*_*g *_to be 0.5.

Here in our case we have taken the general kernel estimator as the pilot estimate of the density f˜
 MathType@MTEF@5@5@+=feaafiart1ev1aaatCvAUfKttLearuWrP9MDH5MBPbIqV92AaeXatLxBI9gBaebbnrfifHhDYfgasaacH8akY=wiFfYdH8Gipec8Eeeu0xXdbba9frFj0=OqFfea0dXdd9vqai=hGuQ8kuc9pgc9s8qqaq=dirpe0xb9q8qiLsFr0=vr0=vr0dc8meaabaqaciaacaGaaeqabaqabeGadaaakeaacuWGMbGzgaacaaaa@2E10@(*X*/*g*).

Now plugging in these above estimates f^
 MathType@MTEF@5@5@+=feaafiart1ev1aaatCvAUfKttLearuWrP9MDH5MBPbIqV92AaeXatLxBI9gBaebbnrfifHhDYfgasaacH8akY=wiFfYdH8Gipec8Eeeu0xXdbba9frFj0=OqFfea0dXdd9vqai=hGuQ8kuc9pgc9s8qqaq=dirpe0xb9q8qiLsFr0=vr0=vr0dc8meaabaqaciaacaGaaeqabaqabeGadaaakeaacuWGMbGzgaqcaaaa@2E11@(*X*_*u*_/*g*) in the equation 2 we get,

P^(g/Xu)=qg⋅f^(Xu/g)∑g′=1kqg′⋅f^(Xu/g′).     (10)
 MathType@MTEF@5@5@+=feaafiart1ev1aaatCvAUfKttLearuWrP9MDH5MBPbIqV92AaeXatLxBI9gBaebbnrfifHhDYfgasaacH8akY=wiFfYdH8Gipec8Eeeu0xXdbba9frFj0=OqFfea0dXdd9vqai=hGuQ8kuc9pgc9s8qqaq=dirpe0xb9q8qiLsFr0=vr0=vr0dc8meaabaqaciaacaGaaeqabaqabeGadaaakeaacuWGqbaugaqcaiabcIcaOiabdEgaNjabc+caViabbIfaynaaBaaaleaacqqG1bqDaeqaaOGaeiykaKIaeyypa0ZaaSaaaeaacqWGXbqCdaWgaaWcbaGaem4zaCgabeaakiabgwSixlqbdAgaMzaajaGaeiikaGIaemiwaG1aaSbaaSqaaiabdwha1bqabaGccqGGVaWlcqWGNbWzcqGGPaqkaeaadaaeWbqaaiabdghaXnaaBaaaleaacuWGNbWzgaqbaaqabaGccqGHflY1cuWGMbGzgaqcaiabcIcaOiabdIfaynaaBaaaleaacqWG1bqDaeqaaOGaei4la8Iafm4zaCMbauaacqGGPaqkaSqaaiqbdEgaNzaafaGaeyypa0JaeGymaedabaGaem4AaSganiabggHiLdaaaOGaeiOla4IaaCzcaiaaxMaadaqadaqaaiabigdaXiabicdaWaGaayjkaiaawMcaaaaa@5D63@

Then the decision rule becomes,

**Assign unit u to class g if ***q*_*g*_·f^
 MathType@MTEF@5@5@+=feaafiart1ev1aaatCvAUfKttLearuWrP9MDH5MBPbIqV92AaeXatLxBI9gBaebbnrfifHhDYfgasaacH8akY=wiFfYdH8Gipec8Eeeu0xXdbba9frFj0=OqFfea0dXdd9vqai=hGuQ8kuc9pgc9s8qqaq=dirpe0xb9q8qiLsFr0=vr0=vr0dc8meaabaqaciaacaGaaeqabaqabeGadaaakeaacuWGMbGzgaqcaaaa@2E11@(*X*_*u*_/*g*) > *q*_*h*_·f^
 MathType@MTEF@5@5@+=feaafiart1ev1aaatCvAUfKttLearuWrP9MDH5MBPbIqV92AaeXatLxBI9gBaebbnrfifHhDYfgasaacH8akY=wiFfYdH8Gipec8Eeeu0xXdbba9frFj0=OqFfea0dXdd9vqai=hGuQ8kuc9pgc9s8qqaq=dirpe0xb9q8qiLsFr0=vr0=vr0dc8meaabaqaciaacaGaaeqabaqabeGadaaakeaacuWGMbGzgaqcaaaa@2E11@(*X*_*u*_/*h*) **for g ≠ h**.

when f^
 MathType@MTEF@5@5@+=feaafiart1ev1aaatCvAUfKttLearuWrP9MDH5MBPbIqV92AaeXatLxBI9gBaebbnrfifHhDYfgasaacH8akY=wiFfYdH8Gipec8Eeeu0xXdbba9frFj0=OqFfea0dXdd9vqai=hGuQ8kuc9pgc9s8qqaq=dirpe0xb9q8qiLsFr0=vr0=vr0dc8meaabaqaciaacaGaaeqabaqabeGadaaakeaacuWGMbGzgaqcaaaa@2E11@(*X*_*u*_/*g*) will be different for these two methods.

In both of these kernel methods we have to estimate some of the parameters, the window function **h **for each class. To simplify the problem we assumed same window function for each class, as because in kernel method this windows are smoothed again, so collective choice is not a bad idea. We selected the smoothing parameters simultaneously by minimization of the cross-validated estimate of the error of classification of the Baye's rule by plugging in these kernel estimates of the group-conditional densities from the knowledge.

It is pleasant to note that maximum probability rule is just a basic concept about modeling, and decision making rule. But it heavily depends upon the above described estimation step which is the heart of the modeling process. With the development of this estimation methods modeling becomes much more flawless and the classification technique becomes stronger. This phenomenon is noted at the time of testing. Adaptive kernel method with its superiority in estimation of tail probability gets an edge over the other methods.

## 7. Classification performance of ANN

The three different bacteria data sets were analyzed using three supervised ANN classifiers: the multilayer perceptron (MLP), probabilistic neural network (PNN), and radial basis function (RBF) network paradigms. Training of the neural networks was performed with 40% of the data set. The remaining 60% of the data were used for testing the neural networks. These percentages were selected arbitrarily and were applied for all data sets. The aim of this comparative study was to identify the most appropriate ANN paradigm, which can be trained with best accuracy, to predict the type of ENT infections or, in other words, the type of ENT bacteria [3,27].

### 7.1 Performance of MLP

A MLP network (with learning rate equal to 0.2 and a momentum term equal to 0.3) with three 3–18 inputs, 2 hidden layers and 3 outputs neurons reached a success rate of 75% in overall classification for three ENT bacteria.

### 7.2 Performance of RBF and PNN

Neurons were added to the network until the sum-squared error fell beneath an error goal (0.000015) or a maximum number of internal neurons was reached upto 50. It is important that the spread parameter should be large enough so that the radial basis neurons respond to overlapping regions of the input space, but not so large that all the neurons respond in essentially the same manner. For both the networks, the spread parameter was set to 1. PNN was able to correctly classify 85% of the response vectors whereas the level of correct classification in the RBF network was up to 92%.

## 8. IBC implementation and results

**Figure 8:**
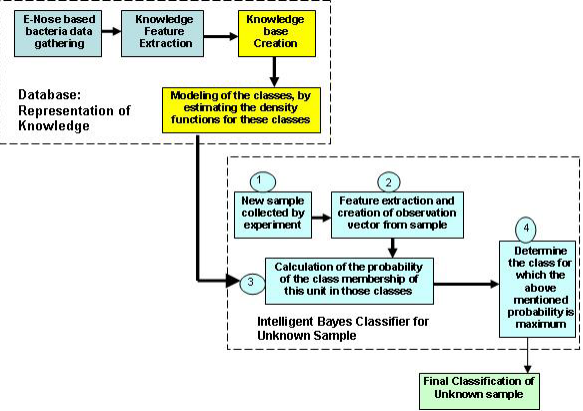
Representative basic block diagram for the implemented unsupervised Bayes classification system [1].

MATLAB [31] implementations have been performed to evaluate these new IBC algorithms on hybrid sensor based e-nose data. Novelty of these algorithms is that there is no need for any supervised neural network training and very rapid classification is performed. Representative basic block diagram for the implemented unsupervised classification system is shown in figure 8.

The best classification system we achieved is based on **'newly extracted feature based knowledge' **and **'Adaptive Kernel Estimator'**. Implemented Bayes classification system was used to classify unknown bacterial samples [1].

*General Algorithm steps*:

• Knowledge base creation based on multiple features.

• Modeling of the classes, by estimating the density functions for these classes.

• New sample collected by experiment.

• Feature extraction and creation of observation vector from sample.

• Calculation of the probability of the class membership of this unit in those classes.

• Determine the class for which the above mentioned probability is maximum.

• Final classification and decision.

The main novelty of this paper is that an innovative knowledgebased Bayes classifier depending upon "Baye's theorem" and "maximum probability rule" has been investigated for these three groups of ENT bacteria. Two different innovative feature extraction techniques, namely **'Kurtosis of the sensory signal'**, and **'Skewness of the sensory signal'**, were developed and tested for the three main bacteria data sets and two sub-strains. It is important to note that there are several different feature extraction techniques are available for e-nose sensory signal processing. The reason behind the selection of these two new feature extraction approaches is that there was not enough consistency in overall classification by considering the most popular techniques, like, 'the difference between the signal's peak and its baseline', 'the area beneath the curve', 'the area beneath the curve left of the peak', and 'the time it takes for the signal to reach its peak'. And also these popular feature extraction techniques based approached produce significant overlapping between data clusters (please see previous section).

For ENT bacteria identification problem, with these two new extracted features and with Adaptive Kernel Estimator we are able to achieve a great level of overall classification consistency.

### 8.1 Classification performance of IBC

To check the ability of classification of IBC over the six different features (the difference between the signal's peak and its baseline, the area beneath the curve; the area beneath the curve left of the peak, the time it takes for the signal to reach its peak, Skewness of the data and Kurtosis of the data) we used the following two testing procedures.

#### 8.1.1 Existing data manipulation method

Randomly we have taken out 20% of the feature points from the existing knowledge base and tried to classify them by considering the remaining 80% feature points in knowledge base as the "present knowledge base". In this way we have tried to classify all the data in the knowledge base and noted the percentage of correct classification for these different classes. Also we aimed to keep the consistency of this testing method along with ANN based testing methods. Advantage of this method is the tested data is virtually behaving like an unknown data. Following this approach we achieved 100% for the overall classification of three species of bacteria, Escherichia coli (E. coli), Staphylococcus aureus (S. aureus), and Pseudomonas aeruginosa (P. aeruginosa) responsible for ear nose and throat (ENT) infections.

In the next stage a sub-classification has been performed using IBC for the classification of two different species of S. aureus, namely MRSA and MSSA. In the later sub-classification problem we also achieved 100% rate of correct classification. All percentages of classification study are based on this our collected data sets at hospital.

#### 8.1.2 Real data based method

To complete this study we decided to take a more realistic approach for testing. So far our classification performance testing was based on existing data sets which originally had been used to develop the knowledgebase. To make the validation of our IBC model in the hospital scenario, we gathered a completely new set of total 60 new patients' data who had infection from three species of bacteria, E. coli, S. aureus (including MRSA and MSSA), and P. aeruginosa.

Kurtosis and Skewness of this unknown data set were extracted as feature and the IBC was used to classify these species of bacteria along with the existing knowledgebase.

Results from these new set of data (completely unknown to our previously developed knowledgebase) were very encouraging. The best results suggest that we are able to identify and classify three bacteria main classes with up to 100% accuracy rate using IBC. We have also achieved 100% classification accuracy for the classification of MRSA and MSSA samples with IBC.

### 8.2 Conclusion

From our results we can conclude following important observations:

• The newly suggested Skewness of the data and Kurtosis of the data features are giving all through better result than the previously used feature in identification of some classes and consistent in other cases.

• A comparative evaluation of the classifiers was conducted for this application. IBC outperformed MLP (75%), PNN (85%) and RBFN (92%). The best percentage of IBC based correct classification is 100%.

• The nonparametric approaches are giving good results but adaptive kernel method is all the way superior to advanced ANN. It speaks for its more accuracy in estimation of tail probabilities of the data distribution. There is a steady increase in the percentage of correct classification when we use IBC in the place of ANN.

• Good results from this study indicate the effectiveness of Bay's maximum probability rule. So it has minimized the total number of misclassification error.

Problem of suitable feature extraction from e-nose sensory data is extensively addressed in this paper where two innovative new scalar feature extraction approaches 'Kurtosis of the sensory data', and 'Skewness of the sensory data' with the old ones as 'area under the curve of sensory data' have been used for extracting representative data. It is also proved that these new feature extraction techniques are giving excellent result for class discrimination in these cases as these methods have been tested on 430 patients' data base. So it can be treated as a major achievement and further research can be carried out in this line.

The kernel methods are the foundation stones of most statistically significant neural network method "Probabilistic neural network". The adaptive kernel method, which is a developed version of this kernel method, can also promote futher works in this direction. IBC could be a significant breakthrough in the field of sensory signal processing of electronic nose technology.

We can conclude that this study proves that "maximum probability rule" and adaptive kernel estimator based IBC can provide very strong solution for identification and very rapid detection of ENT infections in hospital environment.

### 8.3 Discussion

Identification and very rapid detection of ENT infections in hospitals is the most challenging problem worldwide to secure a safe and better hospital environment. Contamination from ENT bacteria in hospital wards is so serious that sometime it's affecting other patients, service nurses and even doctors as well. For example around 40% of cases of S. aureus in the UK are resistant to methicillin and other antibiotics. These types of S. aureus tend to be more common in hospital, because people are more susceptible to infections when they are already unwell. From April to September 2004, 3,519 NHS patients were infected with MRSA. It is estimated that the NHS spends around £1 billion per annum on hospital-acquired infections including MRSA.

At present hospitals use full pathological infustructure for any patients with any type of infection. But long term statistics shows that only 30% of the total infected person are having serious bacterial problem. So there is a need for prioritization. E-nose technology based on IASSP method could be used as a quick screening system to priorities which patients or healthcare workers need more rigorous testing.

With this new electronic nose based technology at least we will be able to exclude 70% of the population of the patients that would be a bonus in terms of the number of people you would want to screen further. Early exclusion of 70% of total patient (who are not suffering from serious ENT infections) would be extremely cost effective, it would be time saving and effectively we will be using less pathological infustructure. This will help us to make a rapid decision and care we would be able to provide much better care that really need it.

## 9. Appendix

### 9.1. Skewness of the data

Skewness is a measure of the asymmetry of the data around the sample mean. If skewness is negative, the data are spread out more to the left of the mean than to the right. If skewness is positive, the data are spread out more to the right. The skewness of the normal distribution (or any perfectly symmetric distribution) is zero. The skewness of a distribution is defined as, y=E(X−μ)3σ3
 MathType@MTEF@5@5@+=feaafiart1ev1aaatCvAUfKttLearuWrP9MDH5MBPbIqV92AaeXatLxBI9gBaebbnrfifHhDYfgasaacH8akY=wiFfYdH8Gipec8Eeeu0xXdbba9frFj0=OqFfea0dXdd9vqai=hGuQ8kuc9pgc9s8qqaq=dirpe0xb9q8qiLsFr0=vr0=vr0dc8meaabaqaciaacaGaaeqabaqabeGadaaakeaacqWG5bqEcqGH9aqpdaWcaaqaaiabdweafjabcIcaOiabdIfayjabgkHiTGGaciab=X7aTjabcMcaPmaaCaaaleqabaGaeG4mamdaaaGcbaGae83Wdm3aaWbaaSqabeaacqaIZaWmaaaaaaaa@39EF@, where *μ *is the mean of x, and *σ *is the standard deviation of x, and E(t) represents the expected value of the quantity t.

### 9.2. Kurtosis of the data

Kurtosis is a measure of how outlier-prone a distribution is. The kurtosis of the normal distribution is 3. Distributions that are more outlier-prone than the normal distribution have kurtosis greater than 3; distributions that are less outlier-prone have kurtosis less than 3. The kurtosis of a distribution is defined as, y=E(X−μ)4σ4
 MathType@MTEF@5@5@+=feaafiart1ev1aaatCvAUfKttLearuWrP9MDH5MBPbIqV92AaeXatLxBI9gBaebbnrfifHhDYfgasaacH8akY=wiFfYdH8Gipec8Eeeu0xXdbba9frFj0=OqFfea0dXdd9vqai=hGuQ8kuc9pgc9s8qqaq=dirpe0xb9q8qiLsFr0=vr0=vr0dc8meaabaqaciaacaGaaeqabaqabeGadaaakeaacqWG5bqEcqGH9aqpdaWcaaqaaiabdweafjabcIcaOiabdIfayjabgkHiTGGaciab=X7aTjabcMcaPmaaCaaaleqabaGaeGinaqdaaaGcbaGae83Wdm3aaWbaaSqabeaacqaI0aanaaaaaaaa@39F3@ where *μ *is the mean of x, *σ *is the standard deviation of x, and E(t) represents the expected value of the quantity t.

### 9.3. Typicality probability

Now P(X/g) denotes the probability that a randomly selected unit has a profile close to X given that the unit is a member of class g, and to be noted that P(X/g) is, in limit, proportional to f(X/g).

### 9.4. Prior probability of class membership

*π*_*g *_is used to denote the "prior probability" of membership in class g, "prior" in the sense that this is a probability of class membership before X_*u *_is known.

### 9.5. Posterior probability of class membership

The probability denoted by P(g/X_*u*_) is the probability of unit u belonging to group g, given that the unit has a particular observation vector X_*u*_, is called the "posterior probability".
